# Impact of history of myomectomy on preterm birth risk in women with a leiomyomatous uterus: a propensity score analysis

**DOI:** 10.1186/s12884-020-03413-w

**Published:** 2020-11-23

**Authors:** Emmanuel Rault, Pierre Delorme, François Goffinet, Aude Girault

**Affiliations:** 1 Maternité Port Royal, AP-HP, Hôpital Cochin, FHU PREMA, F-75014 Paris, France; 2 Université de Paris, INSERM UMR 1153, Obstetrical, Perinatal and Pediatric Epidemiology Research Team (Epopé), Center for Epidemiology and Statistics, FHU PREMA, Université de Paris, 123 boulevard Port Royal, 75014 Paris, France

**Keywords:** Myomas, Preterm birth, Myomectomy, Spontaneous preterm birth

## Abstract

**Background:**

To evaluate if women with a history of myomectomy have a modified preterm birth risk compared to women with myomas during pregnancy.

**Methods:**

Retrospective cohort study including all women with a history of myomectomy (operated group) or uterine myomas during pregnancy (unoperated group) who delivered in a tertiary center between January, 2011 and December, 2017. The operated group included women who had a myomectomy history with or without myomas during the ongoing pregnancy. The unoperated group included women with uterine myoma(s) seen on at least one ultrasound during pregnancy without history of myomectomy. The primary outcome was preterm birth < 37 weeks, and the secondary outcome spontaneous preterm birth < 37 weeks. To control for confounding factors, a propensity score approach was used. Two sensitivity analysis were performed, one repeating the analysis using the propensity score after excluding operated women with persistent myomas and one using a classical multivariable logistic regression model.

**Results:**

The cohort included 576 women: 283 operated women and 293 unoperated women. The rate of preterm birth was similar in the two groups: 12.6% in the unoperated group and 12.0% in the operated group (*p* = 0.82). No difference in preterm birth risk was shown between unoperated and operated women in the cohort matched on the propensity score: OR 0.86; 95%CI [0.47–1.59]. These results were consistent for spontaneous preterm birth (OR 1.61; 95%CI [0.61–4.23]) and for the sensitivity analyses.

**Conclusion:**

In women with a leiomyomatous uterus, a history of myomectomy is not associated with a reduced preterm birth risk.

**Supplementary Information:**

The online version contains supplementary material available at 10.1186/s12884-020-03413-w.

## Background

Myomas are benign uterine smooth muscle tumors affecting 1.5 to 11% of reproductive-aged women [1–4]. Their prevalence during pregnancy is estimated between 1.2 and 12%, depending on the characteristics of the studied population [5, 6].

Previous studies have shown associations between both preterm birth and myomas [2, 6–8] and preterm birth and history of myomectomy [9–14]. Publications concerning the impact of myomectomy on pregnancy outcomes, including preterm birth, do not report any control group. In these studies, the preterm birth rates vary widely from 3 to 38.2% [9–14]. In a retrospective series of 100 women with a history of laparoscopic myomectomy, Dubuisson et al. reported a 14% preterm birth rate [14]. Fukuda et al. who compared the pregnancy outcomes between women operated by laparoscopy and laparotomy reported an overall 11.4% preterm birth rate in a study of 105 women and did not report any significant differences between the two groups [13].

To our knowledge, the preterm birth risk of unoperated women with myomas during pregnancy was never compared to that of women with a history of myomectomy. Therefore, the question of whether myomectomy reduces the preterm birth risk has never been addressed. Yet, the fact that the risk of preterm birth is modified or not by myomectomy is an important information for both surgeons and women with myomas. How myomectomy could reduce the preterm birth risk could depend on the location, the number or the size of myomas [15–17].

Thus, the main objective of this study is to evaluate if women with a history of myomectomy have a modified preterm birth risk compared to women with myomas during pregnancy.

## Methods

We performed a retrospective monocentric cohort using hospital data from January 1st, 2011, to December, 31st, 2017, in a tertiary university hospital maternity unit (Port Royal Maternity, Cochin Hospital, Paris, France).

All singleton pregnancy delivered > 22^+ 0^ weeks during this period were eligible. Multiple pregnancy and women referred from other hospitals after 22 weeks were excluded. Women with myomas or a history of myomectomy were identified using a computer query with a multiple combination of key words: “myoma”, “fibroids”, “myomectomy”, “scared uterus of gynecological origin” and “surgical history”. All medical paper files were reviewed to obtain detailed information from ultrasound and surgical reports.

We included all women with uterine myomas during pregnancy or a history of myomectomy. This study was approved by the National Data Protection Authority (Commission Nationale de l’Informatique et des Libertés, CNIL n° 1,755,849). Under French regulations, this study is exempt from Institutional Review Board (IRB) review because it was an observational study using anonymized data from medical records. Women were informed that their records can be used for the evaluation of medical practices and were allowed to opt out of these studies. The study’s exempt status was confirmed by the Institutional Review Board Ile-de-France.

Gestational age was confirmed during the 1st trimester ultrasound by measurement of the craniocaudal length between 11^+ 0^ and 13^+ 6^ weeks. All ultrasound examinations were performed by licensed sonographers and as recommended in France, at least three ultrasounds were performed during pregnancy. Women with history of myomectomy were managed as women with a history of cesarean delivery i.e. attempted vaginal birth at term or cesarean delivery at 39 weeks if indicated.

We created two groups: “unoperated women”, which included all women with uterine myomas without a history of myomectomy; and “operated women”, which included women with a history of myomectomy, with or without myomas during the ongoing pregnancy. Women could have been operated by either laparotomy, laparoscopy or hysteroscopy. In both groups myomas were considered if there was at least one myoma (operated or not) measuring at least 20 mm or multiple myomas (≥2) whatever the size. The myomas present during the ongoing pregnancy had to be visualized on at least one prenatal ultrasound.

The primary outcome was preterm birth < 37 weeks. The secondary outcome was spontaneous preterm birth < 37 weeks. Spontaneous preterm birth was defined as preterm labor with intact membranes or preterm premature rupture of membranes (PPROM) regardless of labor onset.

We collected the following maternal characteristics: maternal age, body mass index (BMI), geographic origin, socioeconomic status, parity and history of cesarean delivery, previous preterm birth, medical history (preexisting diabetes and hypertensive disorders), and use of Assisted Reproductive Technology (ART). We also collected obstetrical characteristics and outcomes: high blood pressure or preeclampsia, gestational diabetes, small for gestational age, and gestational age at delivery. Myomas’ characteristics were collected from ultrasound and surgical reports: myomas’ location, number of myomas, and size of the largest myoma. If several myomas were present, we reported the location of the closest to the cavity myoma. For example, if a woman presented with a submucosal and serosal myoma it was reported as submucosal.

We compared the baseline clinical and demographic characteristics, the pregnancy characteristics and the myomas’ characteristics between the unoperated and operated women. These comparisons were performed using Pearson χ^2^ tests or Fisher’s tests for qualitative variables and Student t test for quantitative variables.

To identify the variables associated with preterm birth, we performed a bivariate analysis comparing the association between preterm birth and women and myoma characteristics.

To control for confounding factors that might influence both the indication of myomectomy and the occurrence of preterm birth, we used a propensity score approach. A propensity score was estimated for all women by a logistic regression model with myomectomy as the dependent variable. The variables included in the propensity score were variables known in literature as associated with preterm birth [18] or indication of myomectomy [19]. Operated women and unoperated women were matched with a one-to-one nearest neighbor matching algorithm. The matched cohort was then used to evaluate the association between myomectomy and preterm birth. In the matched cohort, odds ratios (OR) and their 95% confidence intervals (95% CI) were estimated using logistic regression. Details on the propensity score construction and matching are provided in *Supplementary information S1*.

The analyses were repeated for our secondary outcome.

Two sensitivity analysis were performed. First, the analysis in the matched cohort was repeated after excluding operated women with diagnosed myomas during the ongoing pregnancy. Then, a multivariable model including the variables known in literature as associated with preterm birth [18] and the variables identified by the bivariate analysis (*p* < 0.10) was performed on the whole population. This model did not include the myomas’ characteristics as in the operated group these myomas had been removed. The adjusted odds ratio (aOR) and their 95% confidence intervals (95% CI) reflecting the association between preterm birth and history of myomectomy adjusted on the selected variables: maternal age, BMI, geographic origin, socioeconomic status, parity and history of preterm birth and use of ART, were assessed through multivariable logistic regression.

All *P* values were two-tailed, and values of *P* < .05 were considered significant. All statistical analyses were performed using Stata (*StataCorp. 2017. Stata Statistical Software: Release 15. College Station, TX: StataCorp LLC*).

## Results

Among the 32,812 women delivered at Port-Royal hospital between January, 1st, 2011 and December, 31st, 2017, 576 (1.7%) women met the inclusion criteria: 293 unoperated women and 283 operated women. In the operated group, 93 (33%) women had persistent myomas during the ongoing pregnancy, with a median number of persistent myoma of 2 IQR [1–3], 183 (64.7%) women had been operated by laparotomy, 49 (17.3%) women by laparoscopy and 51 (18.0%) women by hysteroscopy.

The operated women were significantly older (*p* = 0.04), more frequently multiparous (*p* < 0.01), had had more frequently a previous preterm birth (*p* = 0.02) than the unoperated women (Table 1). They also required more ART (*p* < 0.01). Before surgery, the operated women had significantly more submucosal myomas, more numerous myomas (*p* < 0.01) and a larger mean size of the largest myoma (*p* < 0.01) than unoperated women (Table 2). The median time between myomectomy and date of conception was 3 years, IQR [1.1–5.9].

**Table 1:** Comparison of women’s preexisting characteristics and obstetric characteristics between the unoperated and operated group, *n* = 576

Women’s preexisting and obstetric characteristics	Unoperated group ***n*** = 293 n (%)		Operated group ***n*** = 283 n (%)		***p***
Age (years)					0.04
≤ 35	121	(41.3)	94	(33.2)	
> 35	172	(58.7)	189	(66.8)	
BMI^a^ (kg/m^2^)					0.43
< 25	158	(53.9)	157	(56.1)	
[25–30[	78	(26.6)	80	(28.6)	
≥ 30	57	(19.4)	43	(15.4)	
Geographical origin,					0.16
France	69	(23.6)	72	(25.4)	
Sub-Saharan Africa	114	(38.9)	126	(44.5)	
Other origins	110	(37.5)	85	(30.0)	
Low socioeconomic status^b^,	130	(44.7)	125	(45.8)	0.79
Parity					< 0.01
Nulliparous	204	(68.6)	157	(55.5)	
Multiparous with no history of cesarean	55	(18.8)	16	(5.6)	
Multiparous with history of cesarean	34	(11.6)	110	(38.9)	
History of preterm delivery if multiparous	13	(4.4)	26	(9.2)	0.02
Medical history,					
Diabetes	4	(1.4)	5	(1.8)	0.70
High blood pressure	10	(3.4)	6	(2.1)	0.34
Use of ART^c^	40	(13.7)	80	(28.3)	< 0.01
Gestational morbidities,					
High blood pressure or preeclampsia	33	(11.3)	30	(10.6)	0.80
Gestational diabetes	52	(17.7)	60	(21.2)	0.29
Small for gestational age	26	(8.9)	17	(6.0)	0.19

^a^*BMI* Body Mass Index ^b^ Low socioeconomic status: women needing the help of a social worker during pregnancy ^c^*ART* Assisted Reproductive Technology

**Table 2:** Comparison of myoma characteristics between the unoperated and operated group, *n* = 576

Myomas’ characteristics	Unoperated group ***n*** = 293 n (%)		Operated group ***n*** = 283 n (%)		***p***
Myoma location^a^					< 0.01
Sub-serosal	83	(28.3)	87	(30.7)	
Intramural	136	(46.4)	98	(34.6)	
Submucosal	15	(5.1)	41	(14.5)	
*N/A data*	59	(20.1)	57	(20.1)	
Number of myomas^a^, mean ± SD	2.1	± 1.6	3.3	± 4.1	< 0.01
< 2	133	(45.4)	113	(39.9)	< 0.01
[2–5[	115	(39.2)	71	(25.1)	
≥ 5	17	(5.8)	44	(15.6)	
*N/A data*	28	(9.6)	55	(19.4)	
Size of the largest myoma^a^, cm, mean ± SD	59.0	± 29.1	73.1	± 39.4	< 0.01
< 5	92	(31.4)	57	(20.1)	< 0.01
[5–10[	143	(48.8)	108	(38.2)	
≥10	25	(8.5)	57	(20.1)	
*N/A data*	33	(11.3)	61	(21.6)	

*N/A data*: Non available data ^a^Before surgery for women in the “operated” group

The crude preterm birth rate was not significantly different between the two groups: 12.6% in the unoperated group and 12.0% in the operated group (*p* = 0.82). These results were consistent for spontaneous preterm birth (4.8% in the unoperated group vs. 4.9% in the operated group; *p* = 0.92) and preterm birth < 34 weeks (8.5% in the unoperated group vs. 8.5% in the operated group; *p* = 0.98).

In the operated group, there were no significant differences in preterm birth rates between women with and without myomas during the ongoing pregnancy (respectively 13.9 and 11.1%, *p* = 0.48). Bivariate analyses comparing the association between preterm birth and women’s preexisting, obstetrical and myoma characteristics are detailed in Table S1.

The matched cohort included 386 women. Details on balance between the groups are given by Fig. S1. There was no significant difference in preterm birth risk between operated and unoperated women in the matched cohort: OR 0.86; 95%CI [0.47–1.59]; *p* = 0.64, Table 3. These results were consistent for spontaneous preterm birth, OR 1.61 (reference unoperated women); 95%CI [0.61–4.23]; *p* = 0.34 (Table S2).

**Table 3:** Association between history of myomectomy and preterm birth < 37 weeks (reference = unoperated women); bivariate analysis (*n* = 576) and logistic regression model in the propensity matched cohort (*n* = 386)

Preterm birth < 37 weeks	OR	[95%CI]	***p***
Bivariate analysis	0.94	[0.57–1.55]	0.82
Logistic regression in the propensity score matched cohort^a^	0.86	[0.47–1.59]	0.64

^a^ Propensity score including: myoma’s location, number of myomas, largest myoma’s size, maternal age, BMI, geographic origin, parity, history of cesarean delivery, history of preterm delivery, and use of ART

The sensitivity analysis (excluding operated women with myomas during the ongoing pregnancy) showed no differences in preterm birth risk or spontaneous preterm birth risk between the two groups, respectively OR 0.78; 95%CI [0.38–1.59]; *p* = 0.49 and OR 1.11; 95%CI [0.34–3.57]; *p* = 0.86.

Analysis by multivariable logistic regression models in the whole population provided similar results to propensity score estimates, aOR 0.89; 95%CI [0.52–1.51]; *p* = 0.67.

## Discussion

In this cohort, the risk of preterm birth did not differ between women with a history of myomectomy and unoperated women with myomas during pregnancy. These results were consistent when studying spontaneous preterm birth and when restricting the analysis to women with a history of myomectomy and with no myomas during the ongoing pregnancy.

Most physicians consider the association between preterm birth and myomas to be due to a lack of uterine distension caused by the presence of myomas in the uterine cavity. Because myomectomy, does not improve the rate of preterm birth or spontaneous preterm birth, even when no residual myomas are present during the ongoing pregnancy, we can hypothesize that lack of uterine distension caused by the presence of myomas in the uterine cavity is not the only mechanism impairing pregnancy course.

To explain the persistence of preterm birth risk after myomectomy we can formulate two hypotheses. First, uterine fibrous scars due to myomectomy could restrict the ability of the uterine cavity to distend. Indeed, it has been showed that history of cesarean section increases the risk of preterm birth. In a case control study evaluating the risk factors of preterm birth < 37 weeks during women’s second pregnancy (*n* = 35,983 women), a 2-fold increased risk of preterm birth was reported in women with a history of cesarean section (aOR = 2.20; 95%CI 1.57 to 3.08, *p* < 0.001) [20]. Moreover, myomas affect myofibroblasts, smooth muscle cells, vessel cells, but also the extracellular matrix which is composed of large amounts of collagen, fibronectin and proteoglycans. In myomas, the type of collagen is modified (collagen type I and type III are abundant), fibrils are formed abnormally and are in disarray [21]. This collagen’s modifications restrict the uterus’s ability to distend when the myomas are present during pregnancy but it is also possible that the changes in collagen persist after myomectomy.

A second hypothesis to explain the absence of differences in preterm birth rates in our two groups is the role of hormonal and inflammatory processes. Myomas are indeed hormonal-dependent tumors growing under the influence of both estrogen and progesterone, they are also known to secrete inflammatory factors such as TGF-β3 and other pro-inflammatory cytokines [22]. Deregulation of uterine levels of steroids and pro-inflammatory cytokines that could persist after myomectomy and have been shown to trigger preterm birth [23]. Indeed, these inflammatory processes have shown to increase the rates of placental pathologies and in particular gestational hypertension and preeclampsia which are responsible for induced preterm births [24]. In our cohort, 11% of women had gestational hypertension or preeclampsia which is more than 2 times higher than the general French population [25]. Our hypothesis is that the hormonal and inflammatory environment is different in women with myomas and persists even after myomectomy.

To our knowledge, this is the first study comparing the risk of preterm birth between women with history of myomectomy and unoperated women with myomas during pregnancy. The analyses were performed on a large cohort of 576 women during a 6 years period in a single tertiary maternity center. The number of women with and without a history of myomectomy was similar for each year of our study period, and no significant change in in perinatal care was noted during the study period.

One of the main strengths of this study is the use of the propensity score approach. Indeed, the use of the matched propensity score enabled us to reduce bias due to confounding variables influencing our exposition (myomectomy) but also our outcome (preterm birth). The sensitivity analyses allow us to show that the preterm birth risk in women with history of myomectomy was not only due to the presence of persistent myomas during pregnancy.

Another strength of our study is the available data on risk factors of preterm birth such as previous preterm birth. To avoid a selection bias, as we are a tertiary university maternity unit receiving women with a risk of preterm birth (induced or spontaneous) from surrounding hospitals, we excluded all referred women.

The main limitation of our study is its retrospective design. The myomas’ characteristics were insufficiently reported on ultrasound and surgical reports which affected the rate of missing data. Nonetheless, the myoma characteristics were imputed using multiple imputation-chained equations enabling us to match 67% of our initial population. The propensity score matching allowed us to compare women with similar characteristics, but cannot balance unmeasured characteristics. No data on women delivering before 22 weeks were available, therefore we cannot conclude on the risk of delivery before term of viability.

## Conclusion

In our cohort, there was no difference in preterm birth risk between women with myomas during pregnancy and women who had been operated of their myomas even when no persistent myomas were present during the ongoing pregnancy. Our findings raise questions about the physio-pathological processes resulting in preterm birth in these women. Nevertheless, prevention of preterm birth risk should not in itself be an indication for surgery.

## Supplementary Information

**Additional file 1:** **Table S1.** Bivariate analysis comparing women’s preexisting characteristics, pregnancy characteristics and myoma characteristics between women with a preterm birth < 37 weeks and women with a term birth, *n* = 576. **Table S2.** Association between history of myomectomy and spontaneous preterm birth < 37 weeks (reference = unoperated women); bivariate analysis, logistic regression model in the propensity matched cohort, and sensitivity analyses. Supplementary Information S1. Construction of Propensity score. **Figure S1.** Standardized differences between unoperated and operated women, for the variables included in the propensity score, before (total population) and after matching (propensity score-matched population).

## Data Availability

The detail datasets used and analyzed are available from the corresponding author on reasonable request.

## Abbreviations

ART: Assisted Reproductive Technology
BMI: Body Mass Index
PPROM: Preterm Premature Rupture of Membranes
