# Supplementary material for: Impact of history of myomectomy on preterm birth risk in women with a leiomyomatous uterus: a propensity score analysis
Source: BMC Pregnancy Childbirth. 2020 Nov 23;20:720. doi: 10.1186/s12884-020-03413-w (PMC7686718; doi:10.1186/s12884-020-03413-w)
Supplement: Supplementary file 1 — Additional file 1: Table S1. Bivariate analysis comparing women’s preexisting characteristics, pregnancy characteristics and myoma characteristics between women with a preterm birth < 37 weeks and women with a term birth, n = 576. Table S2. Association between history of myomectomy and spontaneous preterm birth < 37 weeks (reference = unoperated women); bivariate analysis, logistic regression model in the propensity matched cohort, and sensitivity analyses. Supplementary Information S1. Construction of Propensity score. Figure S1. Standardized differences between unoperated and operated women, for the variables included in the propensity score, before (total population) and after matching (propensity score-matched population). [file 12884_2020_3413_MOESM1_ESM.docx]

**Table S1. Bivariate analysis comparing women’s preexisting characteristics, pregnancy characteristics and myoma characteristics between women with a preterm birth <37 weeks and women with a term birth, n = 576**

| **Women’s preexisting and obstetrical characteristics** | Preterm birth  n=71 | | Term birth  n=505 | | *p* | |
| --- | --- | --- | --- | --- | --- | --- |
| **Myomectomy history** | **34** | **(47.9)** | **249** | **(49.3)** | | **0.82** |
| Age (years) |  |  |  |  | | 0.05 |
| ≤ 35 | 34 | (47.9) | 181 | (35.8) | |  |
| >35 | 37 | (52.1) | 324 | (64.2) | |  |
| BMI^1^ (kg/m²) |  |  |  |  | 0.86 | |
| < 25 | 41 | (57.7) | 274 | (54.6) |  | |
| [25-30[ | 19 | (26.8) | 139 | (27.7) |  | |
| ≥ 30 | 11 | (15.5) | 89 | (17.7) |  | |
| Geographical origin, |  |  |  |  | 0.26 | |
| France | 12 | (16.9) | 129 | (25.5) | |  |
| Sub-Saharan Africa | 31 | (43.7) | 209 | (41.4) | |  |
| Other origins | 28 | (39.4) | 167 | (33.1) | |  |
| Low socioeconomic status | 37 | (53.6) | 218 | (44.0) | | 0.13 |
| Parity |  |  |  |  | 0.78 | |
| Nulliparous | 45 | (63.4) | 316 | (62.6) |  | |
| Multiparous with no history of cesarean | 7 | (9.9) | 64 | (12.7) |  | |
| Multiparous with a history of cesarean | 19 | (26.8) | 125 | (24.8) |  | |
| History of preterm delivery if multiparous | 10 | (14.1) | 29 | (5.7) | <0.01 | |
| Medical history, |  |  |  |  |  | |
| Diabetes | 2 | (2.8) | 7 | (1.5) | | 0.36 |
| High blood pressure | 1 | (1.4) | 15 | (3.0) | | 0.45 |
| Use of ART^2^ | 20 | (28.2) | 100 | (19.8) | | 0.10 |
| Obstetrical pathology during pregnancy, |  |  |  |  |  | |
| High blood pressure or preeclampsia | 19 | (26.8) | 44 | (8.7) | | <0.01 |
| Gestational diabetes | 9 | (12.7) | 103 | (20.4) | | 0.12 |
| Small for gestational age | 23 | (32.4) | 20 | (4.0) | | <0.01 |
| Cervical cerclage, | 7 | (9.9) | 23 | (4.6) | | 0.06 |
| Myoma localization, n (%) |  |  |  |  | | 0.07 |
| Serous | 16 | (22.5) | 154 | (30.5) | |  |
| Intramural | 32 | (45.1) | 202 | (40.0) | |  |
| Submucosal | 12 | (16.9) | 44 | (8.7) | |  |
| Number of myoma, mean ± SD | 2.4 | ± 2.2 | 2.7 | ± 3.2 | | 0.31 |
| <2 | 30 | (42.2) | 216 | (42.8) | | 0.90 |
| [2-5[ | 25 | (35.2) | 161 | (31.9) | |  |
| ≥5 | 6 | (8.4) | 55 | (10.9) | |  |
| Size of the largest myoma, cm, mean ± SD | 62.7 | ± 40.8 | 65.9 | ± 34.1 | | 0.57 |
| <5 | 25 | (35.2) | 124 | (24.6) | | 0.16 |
| [5-10[ | 23 | (32.4) | 228 | (45.1) | |  |
| ≥10 | 11 | (15.5) | 71 | (14.1) | |  |

| **Spontaneous preterm birth <37 weeks** | **OR** | **[95%CI]** | ***p*** |
| --- | --- | --- | --- |
| Bivariate analysis, n=576 | 1.04 | [0.48-2.21] | 0.92 |
| Logistic regression in the propensity score matched cohort*, n=386 | 1.61 | [0.61-4.23] | 0.34 |
| * Model adjusted for maternal age, BMI, geographic origin, parity, previous preterm birth, previous cesarean delivery and use of ART | | | |

**Table S2. Association between history of myomectomy and spontaneous preterm birth <37 weeks (reference=unoperated women); bivariate analysis, logistic regression model in the propensity matched cohort, and sensitivity analyses**

**Supplementary Information S1. Construction of Propensity score**

The women’s propensity score was defined as her probability, based on her individual and myoma characteristics, of having a myomectomy. A propensity score was estimated for all women by a logistic regression model with myomectomy as the dependent variable in relation to the following characteristics: myoma’s location (submucosal or intramural versus serous), number of myomas (continuous variable), largest myoma’s size (continuous variable), maternal age (continuous variable), BMI (continuous variable), geographic origin (France, Sub Saharan Africa or other geographical origin), parity, history of cesarean delivery, history of preterm delivery, and use of ART. The variables included in the propensity score were variables known in literature as associated with preterm birth^14^ or indication of myomectomy^15^.

The proportion of missing data on myomas’ characteristics ranging from 9.6% to 21.6%, we decided to impute this missing data using multiple imputation-chained equations. The characteristics of women with missing data were comparable to those with complete cases (data not shown). We calculated a propensity score for each of the 20 imputed datasets and then used an average propensity score for each woman. Operated women and unoperated women were matched with a one-to-one nearest neighbor matching algorithm without replacement on the average propensity score, within a caliper of 0.05^16,17^. Different caliper values were tested, the caliper at 0.5 including more women with an equivalent distribution of propensity score after matching and with standardized differences for each variable ≤ 20% (Figure S1.).

**Figure S1. Standardized differences between unoperated and operated women, for the variables included in the propensity score, before (total population) and after matching (propensity score-matched population).**
